# The Mosquito Immune System and the Life of Dengue Virus: What We Know and Do Not Know

**DOI:** 10.3390/pathogens8020077

**Published:** 2019-06-13

**Authors:** Debica Mukherjee, Sandeepan Das, Feroza Begum, Sweety Mal, Upasana Ray

**Affiliations:** CSIR-Indian Institute of Chemical Biology, 4, Raja S.C. Mullick Road, Jadavpur, Kolkata 700032, West Bengal, India; debicamukherjee@csiriicb.res.in (D.M.); sandeepan@csiriicb.res.in (S.D.); ferozabegum@csiriicb.res.in (F.B.); sweetymal1994@gmail.com (S.M.)

**Keywords:** *Aedes*, immunity, dengue, RNAi, gut-microbiome, AMP

## Abstract

Flaviviruses are largely transmitted to humans by their arthropod vectors such as mosquitoes or ticks. The dengue virus (DENV) is one of the members of the family *Flaviviridae* and is the causative agent of dengue fever. In the mosquito vector, DENV enters through viremic blood meal and replicates in the mid-gut. Newly formed virion particles circulate to various mosquito organs and get transmitted to the next host in subsequent bites. *Aedes aegypti* and *Aedes albopictus* have intricate immune control to allow DENV production at a sub-pathogenic level. In the mosquito, antimicrobial peptides (AMP) and RNA inference (RNAi) are the two main antiviral strategies used against DENV. Apart from innate immunity, mosquito resident microbes play a significant role in modulating DENV replication. In this review, we discuss different immune mechanisms and preventive strategies that act against DENV in two of its vectors: *Aedes aegypti* and *Aedes albopictus*.

## 1. Introduction

Vector-borne diseases such as yellow fever, dengue fever, etc. are known for their chronic effects on human health. These diseases are spread by vectors such as mosquitoes and other arthropods. Mosquitoes are known to be an important vector of virus transmission. Geographically, tropical and subtropical areas are the most suitable regions for optimal growth of mosquitoes and thus mosquito borne diseases pose great risk the world over.

The dengue virus (DENV) is a single-stranded, positive-sense RNA virus belonging to the *Flaviviridae* and is transmitted to humans and other primates by *Aedes* mosquitoes, importantly *Aedes aegypti* (*Ae. aegypti)* and *Aedes albopictus (Ae. albopictus)*. In humans, DENV infection causes dengue fever (DF) and in severe cases, dengue hemorrhagic fever (DHF) or dengue shock syndrome (DSS). In the transmission cycle, upon injection into the human body, the virus gains entry into the host’s cells, hijacks the host’s cell machinery to replicate, and escapes the host’s immune strategies to elicit pathology symptoms [1]. Conversely, in the mosquitoes, the virus replicates, but it is under strong immune control so that the vector’s life span is not compromised. DENV can inhibit mosquito immunity to an extent that allows it to multiply to a non-pathogenic level [2]. Thus, a fine immunological balance exists in the mosquito so that the virus can persist and multiply.

A female mosquito needs to feed on vertebrate blood before egg laying for egg maturation. The virus enters the mosquito’s system via viremic blood and infects the midgut epithelial cells. It replicates in the midgut cells and then gets released into the hemocoel and disseminates to other tissues via hemolymph, ultimately reaching the salivary glands to infect fresh hosts in subsequent bites.

**Figure 1:**
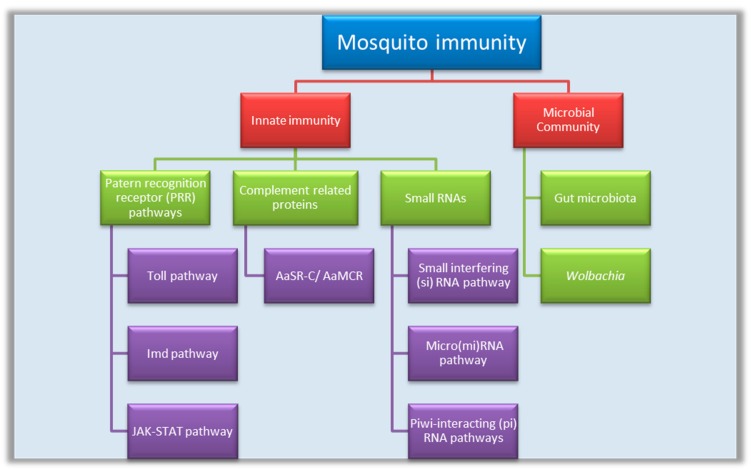
Mosquito immune strategies against the dengue virus (DENV). JAK-STAT = Janus kinase-signal transducer and activator of transcription; AaSR-C = *Ae. aegypti* homolog of scavenger receptor-C; AsMCR = *Ae. aegypti* macroglobulin complement related factor.

In the mosquitoes, innate immunity and a resident microbiome work together against DENV. This review summarizes the molecular mechanism of immune function in the mosquito system against DENV pathogenesis (Figure 1).

## 2. Innate Immunity

The mosquito’s innate immunity includes three main strategies:

1. *Antiviral signaling pathways*: Toll-like pathway, immune deficiency (IMD), and Janus kinase-signal transducer and activator of transcription (JAK-STAT) pathway.
2. *Complement-like proteins*: These include Thioester-containing proteins (TEP); *A. aegypti* macroglobulin complement related factor (AaMCR) and *A. aegypti* homolog of scavenger receptor-C (AaSR-C).
3. *Small RNAs*: These include small interfering RNA (siRNA), micro RNA (miRNA) (derived from the virus), and PIWI-interacting RNA (piRNA).

## 3. Antiviral Signaling Pathways

**Toll-like pathway**: In *Ae. Aegypti*, dorsal ortholog Rel1 and Relish ortholog Rel2 act as Toll and IMD pathway components, respectively, and induce expression of antimicrobial peptides (AMPs) [3]. It was observed that ten days post infection with DENV, the oxidative damage preventative enzymes are suppressed, but Toll and JAK-STAT pathway effectors along with pathogen recognition receptor (PRR) expression are up-regulated [3]. The Toll pathway is a powerful anti-dengue defense system for *Ae. aegypti*, as well as against multiple DENV serotypes [4].

**Figure 2:**
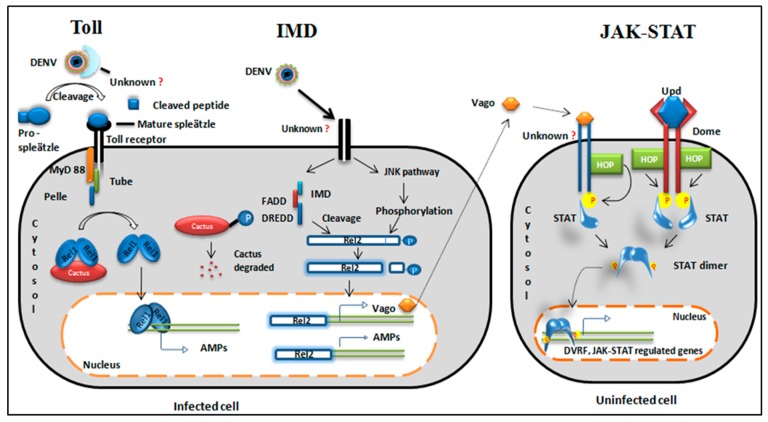
Evolutionarily conserved signaling pathways. *Toll pathway:* The virus is recognized by an unknown cytoplasmic receptor. Cytokine pro-spleatzle is cleaved to active cytokine spleatzle and binds to the Toll receptor. Adaptor proteins are recruited to the Toll receptor. A negative regulator of cactus degrades and a free Rel1 dimer translocates to the nucleus. The Rel1 dimer acts as a transcription factor for Toll regulated genes and produces antimicrobial peptides (AMPs) (cecropin and defensin). *IMD pathway:* The virus binds to a transmembrane receptor of the cell and splits the pathway into two segments. One segment activates the JNK pathway and phosphorylates Rel2. Another segment recruits IMD and other adaptor proteins {Immune deficiency (IMD), fas-associated death domain (FADD) and death related ced-3/Nedd2-like protein (DREDD)} to cleave the C-terminal phosphorylated domain of Rel2.11. Cleaved Rel2 translocates to the nucleus and transcribes AMPs and a secretory protein, Vago. *JAK/STAT pathway:* This pathway is activated either by cytokine-like secretory protein from an infected cell or by the conserved JAK-STAT ligand Upd. Vago is secreted from nearby infected cells, binds to an unknown receptor, and recruits hopscotch (HOP) kinase. Similarly, Dome receptors bind Upd and receptor phosphorylation occurs through HOP kinase. The phosphorylated receptor is a docking site for STAT. STAT phosphorylation leads to dimerization. STAT dimer translocates to the nucleus and transcribes JAK/STAT-regulated genes and dengue virus restriction factor (DVRF).

Toll receptor activation requires virus-derived ligand binding to unknown extracellular PRRs and proteolytic cleavage of pro-spaetzle to activate the cytokine spaetzle, which is the ligand for the transmembrane receptor of the Toll pathway [5]. Spaetzle activates the Toll receptor by cross-linking of receptor ectodomains followed by the relay of the signal through adaptor proteins (Figure 2). 

**Immune deficiency (IMD) pathway**: The immune deficiency pathway operates by virus-receptor binding followed by recruitment of adaptor proteins. In *Drosopila*, the IMD signaling pathway activates an NF-kβ transcription factor, Relish, whereas in mosquitoes, Relish ortholog Rel2 acts as a transcription factor. The IMD pathway in mosquitos gets activated when a virus binds to an unknown receptor [6], which recruits various adaptor proteins. Henceforth, the pathway has two segments as described in Figure 2, one of which activates the Janus kinase (JNK) signaling that phosphorylates Rel2 [7,8], the other part recruits IMD, FADD (fas-associated death domain), and DREDD (death related ced-3/Nedd2-like protein) proteins to cleave the phosphorylated Rel2 [8]. Cleaved Rel2 eventually transcribe IMD related AMP genes (Diptericin and Cecropin) and a signaling molecule called Vago, which is known to have an antiviral role against West Nile virus and *Drosophila* C virus [9,10]. Vago is secreted from the infected cell and acts as a ligand for the JAK-STAT pathway in the neighboring cells (Figure 2). 

DENV infection significantly up-regulates the expression of cecropin-like AMPs [11]. Another study revealed that activation of the IMD pathway by inhibiting the negative regulator (Casper) have no effect on DENV in the midgut of a susceptible *Ae. Aegypti* strain [3,12] whereas in a refractory *Ae. Aegypti* strain, blocking IMD pathway results in an increase in viral replication [12].

In *Drosophila*, IMD is linked to a protein complex (TAK1/TAB2) via a polyubiquitin chain. The TAK1/TAB2 complex leads to the activation of one segment of the IMD pathway [13]. However, in mosquitoes, the Ubiquitine variant (Ub3881) lacks one of the essential lysine residues. Hence, Ub3881 possibly has other residues that target the DENV envelope protein for degradation and down-regulate the production of infectious virus particle [14,15].

The JAK-STAT pathway is an essential defense pathway for anti-dengue immunity in invertebrates [11,16]. In *Ae. aegypti*, the JAK-STAT pathway is activated by different ligands. Unpaired (Upd) is the common ligand that binds to its receptor Dome. Receptor dimerization leads to activation of receptor-associated JAK and downstream signaling (Figure 2). 

According to Souza-Neto et al., mosquitoes become more susceptible to DENV infection if the receptor Dome or JAK homolog HOP is suppressed by RNA inference (RNAi). On the other hand, DENV resistance increases if the negative regulator of the JAK-STAT pathway, protein inhibitor of activated STAT (PIAS) blocks the signaling [16]. The effector genes of DENV are named dengue virus restriction factors (*DVRFs*). DVRF 1 is the transmembrane receptor of the pathway and DVRF 2 recognizes the virus by the antifreeze and allergen domains. The allergen domain has been reported in *Anopheles gambiae* pattern recognition receptor (PRR) MDL1 immune gene. MDL1 immune gene is known to have anti-plasmodium activity. Hence, DVRF2 could be a PRR and be involved in DENV recognition [16].

Although these main signaling pathways restrict viral propagation to non-pathogenic levels, DENV multiplies and accumulates in salivary glands, making the vector a competent virus transmitter.

## 4. Organ-Specific Antiviral Strategies

**Figure 3:**
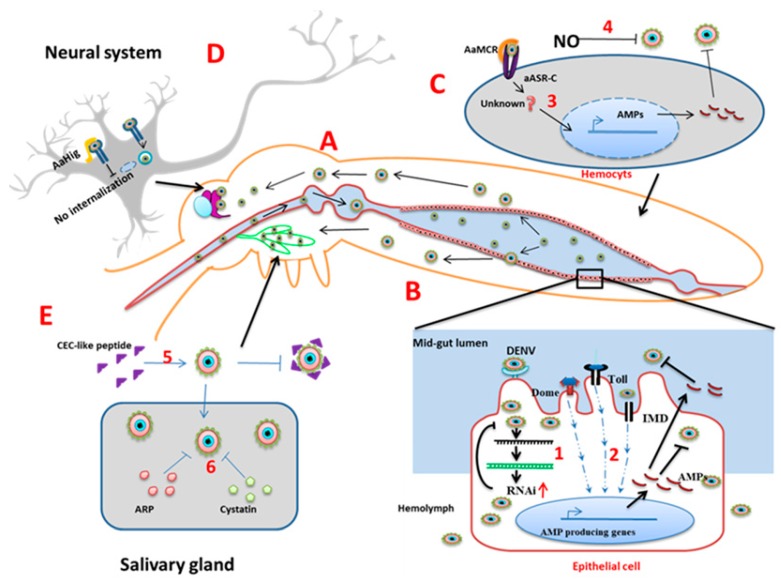
Organ-specific mosquito antiviral strategies. (**A**): In the *Ae. aegypti* system, DENV enters through blood meal and replicates inside the mid-gut. From the mid-gut, DENV is released to the hemolymph, salivary glands, and brain. (**B**): Inside the mid-gut epithelial cells, (1) RNA interference limits viral replication and (2) immune signaling pathways generate AMPs that block the virus inside the cells and also diffuse out to the hemolymph. (**C**): In the hemocytes, (3) complement-like factor AaMCR and its scavenger receptor AaSR-C interact with the signaling AMP production. (4) Phenoloxidase (PO) encapsulates DENV in the hemolymph. (**D**): In the neurons, AaHig binds to the envelop protein of the DENV as well as the cell membrane to block endocytosis. (**E**): In the salivary glands (5) extracellular cecropin-like peptides inhibit the virus and (6) intracellular cystatin and ankyrin repeat-containing protein (ARP) limit virus production.

**Mid-gut**: The mid-gut (Figure 3A) is the initial tissue that comes into contact with the virus-containing blood meal. The first line of defense in the mid-gut are physical barriers such as the mid-gut infection barrier (MIB) and mid-gut escape barriers [17,18,19,20]. The mid-gut infection barrier may form due to the lack of entry receptors on the epithelial cells [17] or pathogen compartmentalization by the peritrophic matrix [18]. After successful entry of the virus inside midgut cells, uncoating, replication, and new virus particle assembly occurs. If the newly formed virions are not able to cross the basal lamina of the epithelial cells to spread in the hemolymph or are unable to infect secondary organs, the prevention of these events are referred to as midgut escape barriers (MEB) [19]. In *Ae. aegypti*, the innate immune signaling pathways become active during DENV infection. Exogenous siRNA pathways work against viral infection in the midgut (Figure 3B) [21]. Additionally, the gut microbiome plays an important role (discussed later). 

**Hemolymph**: From the mid-gut, DENV is released into the hemocoel, then disseminated to other organs. In hemolymph, hemocytes allow virus replication, but innate immunity limits the distribution. Host nitric oxide (NO) inhibits DENV replication in hemocytes. NO production increases during infection and exogenous NO reduces the DENV replication level (Figure 3C) [22]. 

**Complement related proteins:** Another defense mechanism in the hemolymph, which controls flaviviral infection, comprises the complement-related proteins. The mosquito genome encodes a thioester-containing protein (TEP) that recognizes flaviviruses and leads to the expression of an antimicrobial peptide [23]. *Ae. aegypti* macroglobulin complement related factor (AaMCR) belongs to the insect TEP family, which recognizes viral particles. The *Ae. aegypti* homolog of the Scavenger receptor C (AaSR-C) mediates the binding of AaMCR to the DENV particles [24]. Thus, when expression of AMPs in the hemocytes is up-regulated, AMP diffuses to the hemolymph and plays an anti-dengue role (Figure 3C). 

**Salivary glands (SGs):** Salivary glands contain the major pool of the virus before transmission. Active replication of the DENV occurs leading to a high virus titer. In the SGs, the virus again has to pass through salivary gland infection and escape barriers (SGIB and SGEB), which are the ultimate restriction points before virus transmission. The SGIB may form due to the lower amount of virus titer in the hemolymph [25] or the presence of the basal lamina of the SG cells, which hide cellular entry receptors [26]. The SGEB may result because of incomplete apoptosis of the SG acinar cells, which is required for the virus to release via saliva [27]. The DENV induces multiple immune effectors in the salivary glands. In *Ae. Aegypti*, Toll and IMD pathways are activated and this gives rise to a putative antibacterial peptide, cecropin-like peptide (CEC-like peptide) (Figure 3E), which shows anti-DENV activity [11]. Other immune regulatory genes that are upregulated in a DENV infection include the Toll pathway receptor and adaptor proteins (Toll5A and MYD88). However, a peptide from the Defensin family is markedly downregulated [11].

In the SGs, complement-like factors AaMCR and AaSR-C have a role as antivirals that mediate virus recognition and AMP production [24]. Putative cystatin (CS) gene expression and ankyrin repeat containing protein (ARP) gene expression also increase in the DENV infection along with 12 other immune modulator genes (Figure 3E) [28]. Silencing of these genes results in an increased viral load, which indicates their role in antiviral immunity.

**Neuronal system:** The neuron-specific resistance mechanism of mosquitoes against viral infection is largely underexplored. In mosquito brains, an immune factor named *Ae. aegypti* homolog of *Hikaru Genki* (*AaHig*) is expressed ubiquitously [29]. The Aahig protein contains the conserved immunoglobulin domain and complement control protein domain (CCP) (Sushi domain) [29]. The AaHig protein specifically localizes at membranes of the neural cells and its CCP domain interacts with the surface envelope proteins of the flaviviruses. The binding of AaHig to the virus envelope directly blocks viral entry (endocytosis) (Figure 3D). 

**Antimicrobial peptides/proteins:** Antimicrobial peptides/proteins (AMPs) are the main components of the humoral immune response and are synthesized in fat bodies, hemocytes, and epithelial cells in response to DENV infection [24]. AMPs are secreted into hemolymph, distributed to different organs, and display antimicrobial activity against a broad spectrum of microorganisms. In response to microbial invasion, multiple signaling pathways get activated such as the IMD, Toll, and JAK-STAT pathways, with the JAK-STAT pathway being the most important in the case of viral infection [16]. These signaling events lead to production of AMPs that are the important immune effectors. AMPs have also been shown to be important for maintaining mosquito gut immunity [3]. Seventeen AMPs have been discovered in *A. aegypti* and are classified into five independent groups: cysteine-rich defensins, alpha helical peptides cercopins, cysteine-rich peptides gambicins, glycine-rich peptides attacins, and diptericins. **Defensins** are the predominant AMPs of the *Aedes* mosquitoes, the production of which is induced in response to infection by bacteria, filarial worms, and viruses [30]. Defensins are cysteine-rich in nature [31] and have been shown to function by attaching to cell membranes, leading to pore formation, which causes efflux of various essential nutrients [32]. DENV-2 infection-induced defensin expression in C6/36 cells was enhanced with increased viral multiplicity of infection (MOI). Defensins A, C, and D were found to increase due to DENV infection [24]. Cecropins are small proteins which inhibit proline uptake, causing leaky membranes. Cecropins have several isoforms and many of them were found to increase during DENV infection. They can lyse cellular membranes in the case of bacteria and are also capable of inhibiting proline uptake and inducing membrane leakage [33,34]. A 59 amino-acid peptide (AAEL000598) ceropin isolated from the salivary glands of *Ae. aegypti* showed antiviral activity against both the DENV and Chikungunya viruse [7]. Cercopin P1 has been shown to inhibit release of viral particles [35]. Gambicin is another AMP found in mosquitoes and other insects, the mature form of which is a 61 residue peptide having eight cysteins connected with four disulfide bridges. Gambicin expression increases by more than two-fold during a DENV2 infection of *Ae. aegypti* [24]. Attacin is a 20 kD protein that acts by inhibiting biosynthesis of the outer membrane proteins in gram-negative bacteria [24]. Attacin mRNA levels increase due to DENV infection in mosquitoes. The same study reported a slight increase of yet another AMP called ‘diptericin’ during DENV2 infection in *Ae. Aegypti*.

Although the expressions of defensin, cecropin, and gambicin increase during viral infection in mosquitoes and other insects, their mechanism of action is still unclear and needs further research. In contrast, the other two AMPs, namely attacin and diptericin, have been shown to exert an antiviral response in *Drosophila* by controlling viral RNA synthesis during Sindbis virus (SINV) infection, and knocking down these genes increases the viral load in *Drosophila* [36]. Although the importance of AMPs in mosquito immunity is now an established fact, technical difficulties in the isolation of hemocytes (AMP production site) and the unavailability of suitable cell lines have made the characterization of the molecular mechanisms of AMP function challenging. The importance of AMPs as immune effectors and antiviral agents warrants detailed investigation of their molecular mechanisms of action.

## 5. Small RNA Mediated Immunity

**Figure 4:**
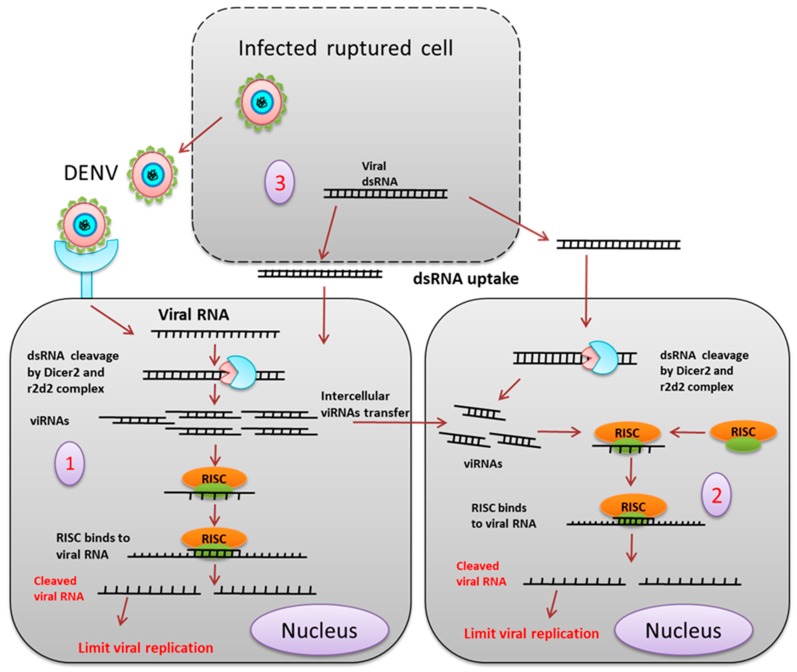
siRNA mediated pathway. (1) The exogenous siRNA pathway is activated when the virus-derived dsRNA is recognized and cleaved by the Dcr2 and r2d2 complex into about 19-21 bp long siRNA. siRNAs duplexes are loaded onto the RISC (RNA induced silencing complex), which degrades the passenger strand (2). The RNAi signal or the siRNA from the virus-infected cell enters the neighboring uninfected cell through a gap junction or cytoplasmic bridge (3). Viral dsRNA released from the infected ruptured cell is taken up by the cells, which provides adaptive immunity by the siRNA pathway.

**siRNA**: Small interfering RNA (siRNA) is one of the important components of the RNAi mechanism (Figure 4). Gaines et al. showed that infection of C6/36 cells with a double subgenomic Sindbis (dsSIN) virus-carrying precursor of the membrane (prM) coding region of the DENV2, provides resistance against DENV2 but not DENV3 and DENV4 [37]. Northern blot and immunofluorescence confirmed the presence of sense and antisense prM RNA and prM proteins, respectively, in the infected cells. RNAi as an innate antiviral immunity mechanism against the DENV was confirmed when expression of untranslatable prM in C6/36 cells showed resistance against DENV2. Expressing dsRNA derived from the genome of the DENV in C6/36 cells restricts DENV2 replication [38]. Intrathoracic injection of a dsSIN virus containing the prM of DENV2 in *Ae. aegypti* restricted DENV2 RNA accumulation in the head tissue, salivary glands, and midgut [39]. DENV2 serotype-specific vsiRNAs are generated upon infection in *Ae. Aegypti*, and knockdown of the siRNA pathway components dcr2, Ago2, and r2d2 leads to an increase in viral replication and shortening of the extrinsic incubation period [21]. 

It was found that DCR2 and AGO2 transcript levels were significantly increased in the midgut with DENV infected blood meal in the early days of infection, but the levels were equalized in later stages [40]. Direct transfection of synthetic siRNA against the membrane glycoprotein precursor gene of DENV1 in C6/36 cells reduced the viral load and increased cell survival rate [41,42].

Previously, it was thought that the siRNA mediated RNAi against the virus by a local and cell-autonomous phenomenon, but recent studies have shown that the RNAi signal can pass from one cell to another. In insects, the virus-derived siRNA can pass from an infected cell to a non-infected cell via gap junctions or cytoplasmic bridges, thereby transferring the RNAi response to neighboring cells. 

**Figure 5:**
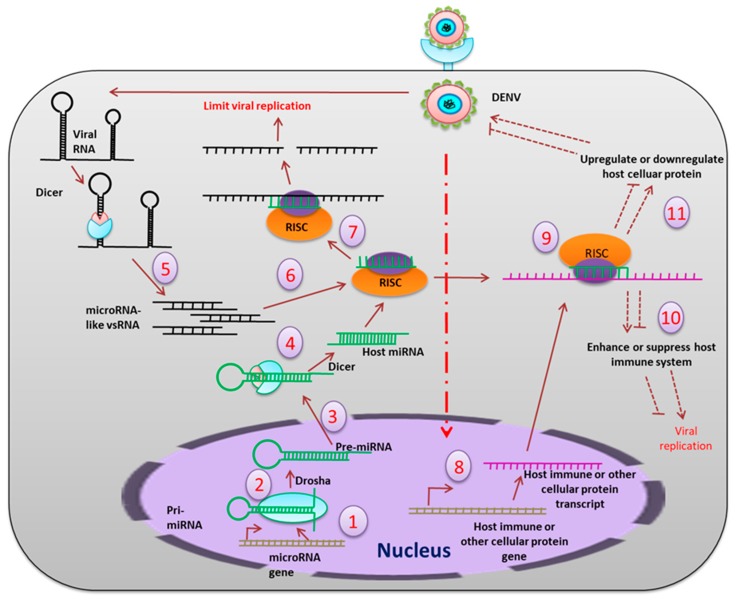
The miRNA pathway mediated inhibition of viral replication. Primary miRNAs (pri-miRNAs) are transcribed (1) from the host genome, which is cleaved (2) by the nuclear resident Drosha into approximately 70 bp-long precursor miRNAs (pre-miRNAs), which then are transported (3) to the cytoplasm, where they get further cleaved (4) by Dicer into mature 21-23 bp-long miRNA. On the other hand, the viral RNA, which forms hairpin secondary structures, is also cleaved (5) by the Dicer to generate microRNA-like viral small RNA, which are further loaded (6) into RISC. RISC complex blocks (7) the viral replication directly by interacting with the single stranded viral RNAs. Viral infection also influences production of host immune transcripts and other cellular transcripts (8). Translation of these transcripts is also modulated (9) by the host miRNAs or microRNA-like viral small RNAs. This results in up-regulation or down-regulation of the immune molecules (10) and other host cellular proteins (11), which may positively or negatively regulate viral replication in the host cell.

**miRNA:** In the mosquito, miRNAs can interact with RNA viruses either directly or indirectly (Figure 5), where the direct interaction involves binding of the miRNA to the viral RNA genome, and the indirect interaction introduces a virus-mediated change in the host transcriptome [43]. In a time-dependent miRNA expression profile study in *Ae. aegypti*, it was shown that at 2–4 days post-exposure (dpe), five miRNAs were modulated, but this increased to twenty-three at 9 dpe. In silico analyses revealed 464 gene targets when miRNA bound to the 3′ untranslated region (UTR). These genes include those expressing proteins involved in transport, transcriptional regulation, mitochondrial function, chromatin modification, and signal transduction processes that help in viral replication and dissemination. A number of endogenous miRNAs are induced after blood feeding in *Ae. aegypti* (e.g., aae-miR-375). The targets of aae-miR-375 include two immune-related genes, *cactus* and *REL1*. *Cactus* is upregulated by aae-miR-375, whereas *REL1* is downregulated. The aae-miR-375 also enhances DENV2 replication during infection [44]. In *Ae. albopictus*, sixty-six differentially expressed miRNAs were identified during DENV infection. Among these, miR-34-5p, which was upregulated, targets the Toll pathway signaling protein (REL-1), and the peptidoglycan recognition protein LE and AMP defensin D. miR87, which were down-regulated, target the Toll pathway [45].

During persistent viral infection, the miRNA expression profile changes [46]. Some of miRNAs that are upregulated are miR-927, miR-87, miR-210, miR-2a3p, miR-190, and miR-970 whereas the miRNAs that are downregulated include miR-252, miR-263a-3p, miR-92b, miR-10-5p, miR-9a-5p, miR-9a-1, miR-124, miR-286a, and miR-286b [46]. Studies of the targets for these miRNAs revealed target proteins to be those involved in ubiquitination, innate immune response, oxidative stress response, cytoskeletal maintenance, fatty acid biosynthesis, intracellular protein transport, exocytosis, autophagy, and pH regulation [46]. Modulation of protein expression by miRNAs helps in maintaining an equilibrium between viral replication and host antiviral response during persistent infection.

MicroRNAs like viral small (vs) RNAs are produced from the viral genome. Twenty-three microRNA-like vsRNAs were identified and were found to originate from the 5′UTR and 3′UTR of the DENV2 in the argonaute 2 (AGO2) dependent pathway [47]. Among those, DENV-vsRNA-5 was found to inhibit DENV2 replication by targeting the non-structural protein-1 (NS1). Recently, researchers have been trying to develop transgenic mosquitoes that are resistant to DENV infection and transmission. A genetically engineered *Ae. aegypti* was developed, in which artificial antiviral miRNA genes were introduced under the polyubiquitin promoter targeting the DENV3 non-structural proteins NS2B, NS3, and NS5. This reduced the DENV3 transmission rate effectively [48].

**Figure 6:**
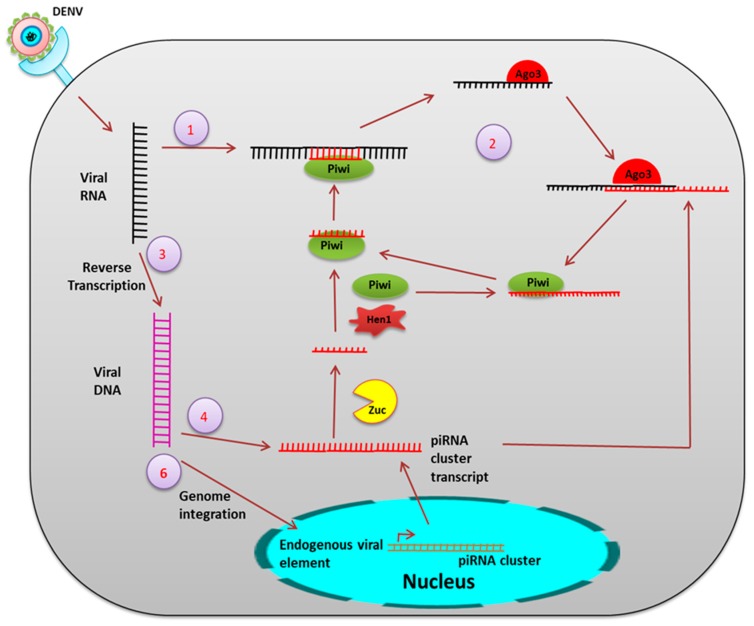
The piRNA mechanism. During viral infection, the ss viral RNA is processed by PIWI proteins [56] (1), PIWI5 and PIWI6, to form the primary piRNA. This primary piRNA may undergo a ping-pong amplification loop (2) to produce Ago3-dependent secondary piRNA. Additionally, viral RNA is also reverse transcribed to form viral DNA (vDNA) (3). These vDNA derived transcripts serve as additional precursors for the viral piRNA production (4,5). Genomic integration of vDNA might occur. (6) Integration in the germline leads to formation of endogenous nonretroviral element.

**piRNA**: piRNAs are 24–30 nucleotides long, small RNA formed from the intergenic region termed a piRNA cluster and are processed from the single-stranded piRNA precursor (pre-piRNA) via a dicer-independent mechanism [49] (Figure 6). 

In *Drosophila melanogaster*, pre-piRNAs are trimmed at both the 5′ and 3′ ends by mitochondria-associated nuclease Zucchini (Zuc) [50] and by an unknown 3′-5′ exonuclease [51]. The piRNAs are loaded onto PIWI proteins before 3′ trimming and are 2′-O-methylated at the 3′ end by a methyltransferase, DmHen1/Pimet, forming mature PIWI-piRNA complexes [52]. Aub- and PIWI-bound primary piRNAs are antisense and uridine (1U) biased at the 5′ end. AGO3 binds to these piRNAs to form the piRNA-induced silencing complexes (piRISCs) which are transported to the nucleus to cleave complementary target transcripts. This process generates a new secondary sense piRNA that pairs with the antisense piRNA precisely by 10 nts at the 5′end. This secondary piRNA specifically contains adenosine at position 10 (10A°) and again undergoes the same process of 2′-O-methylation at the 3′ end followed by AGO3 binding to generate Aub-bound piRNAs. This piRNA amplification loop is referred to as the “ping-pong” cycle [53,54].

The piRNAs play a role in the innate antiviral response in insects. Virus infection induced piRNAs were first observed in *Drosophila* ovarian somatic sheet (OSS) cells [42]. In a small RNA profile in C6/36 cells after DENV infection, a high read peak was found in 27 nts-long RNAs and these viral small RNAs were positive sense [55]. Dicer2 activity was found to be significantly very low in C6/36 cells, indicating the possibility of an alternate mechanism for production of these 24–30 nts-long vsRNAs. A time-dependent small RNA profiling of DENV infected *Ae. aegypti* showed that 24–30 nts-long RNA reads were high at two days post infection (dpi) but these read percentages decreased after 9 dpi. These 24–30 nts-long RNAs were considered as viral piRNA (vpiRNA) and were slightly 10A biased, but had no preference for uridine at the 5′extreme as seen in other piRNAs [41]. In a comprehensive analysis of both viral and host-derived small RNAs in DENV2 infected *Ae. aegypti* Aag2 cells, all three types of small RNAs were identified, which include vsiRNAs, host miRNAs, and viral piRNAs. Knockdown of PIWI5 and Ago3 resulted in the reduction of these vpiRNA levels, confirming that *Aedes* PIWI proteins are associated with the production of DENV-derived vpiRNAs [56]. Both vpiRNAs and virus-induced host endogenous piRNAs (vepiRNAs) produced in DENV2 infected *Ae. albopictus* were found to be confined to specific hot spot regions in the DENV-2 genome, especially in the NS5 gene region [57].

Endogenous viral elements derived from non-retroviral RNA viruses have been identified in different mosquito species. In *Ae. aegypti* and *Ae. Albopictus*, a large number of non-retroviral integrated RNA viruses (NIRVS) were identified including flaviviruses [58]. NIRVS were associated with the piRNA cluster. Three endogenous flaviviral elements (EFVE) identified from *Ae. albopictus* cell lines and one from *Ae. aegypti* cell lines produce 27 to 29 bases-long small RNAs, which were 1U biased and antisense to the viral open reading frame (ORF). DENV infection did not affect the EFVE-derived transcript level, which also suggests constitutive expression. PIWI proteins were found to be associated with the production of this EFVE-derived small RNA. This indicates a complex NIRVS-derived small RNA-mediated antiviral defense in mosquitoes.

## 6. Microbial Community

**Role of gut microbiomes:** The gut microbiome forms a complex ecological environment and can influence vector competence in various ways. In a gut microbiome study, 40 different types of bacteria were isolated from the gut of *Ae. aegypti* [59]. These bacteria upregulated antimicrobial peptide gene transcription. *Chromobacterium sp. (Csp_P)* were isolated from the midgut of field-collected *Ae. aegypti*. *Csp_P* showed entomopathogenic activity, as its exposure to larval breeding water and ingestion by adult mosquitoes reduced survival of both the larvae and adult. During *Csp_P* colonization, cecropin E and G and defensin C displayed at least a two-fold increase in transcript abundance in the midgut. Colonization of *Csp_P* in the midgut also inhibited DENV infection in *Ae. aegypti* [60]. Very little is known about the gut mycobiome, and only a few members of fungi have been characterized. *Talaromyces (Tsp)* was isolated from the gut of field-caught *Ae. aegypti* and found to render mosquitoes more permissive to DENV infection. 

The *Talaromyces* (Tsp) secretome was found to have a profound modulating effect on the midgut transcriptome. It downregulates trypsin encoding genes involved in blood digestion and also reduces trypsin enzymatic activity, which may play a role in the promotion of DENV infection in the midgut [61].

***Wolbachia*mediated defence:** Dengue infection in *Aedes* mosquitoes has been reported to be suppressed in the presence of *Wolbachia* species, gram negative endosymbiotic bacteria [62]. DENV-2 dissemination to secondary organs is inhibited by *Wolbachia* in *Ae. albopictus* [63]. In spite of being a natural host of *Wolbachia*, *Ae. albopictus* becomes a competent vector for the DENV because of the restricted tissue tropism of the wFLu *Wolbachia* strain in *Ae. albopictus*. In the case of *Aedes aegypti*, it is transinfected with *Wolbacia* strains wMel and wMelPop, which makes the mosquitoes resistant to DENV infection [64]. These two strains show organ-specific restriction with wMelPop giving strong resistance towards DENV in the mid-gut tissue and salivary glands while wMel acts on salivary glands only. Another study also reported the absence of DENV-2 particles post infection in female *Ae. aegypti* with wMel [65].

Although the mechanism of virus resistance by the *Wolbachia* species is not fully understood, possible mechanisms include competition for host resources, indirect connection to immune signaling pathways, and reactive oxygen species (ROS) production. A recent study revealed that DENV-derived subgenomic flaviviral RNA (sfRNA) inactivates cellular exoribonuclease (XRN1) in the absence of *Wolbachia*, thereby giving stability to viral RNA. In the presence of *Wolbachia*, XRN1 remains inactivated and RNA degradation increases among all four DENV serotypes [66]. 

## 7. Lipid Droplet (LD) and Immunity

Lipid droplets (LDs) are structures composed of a fatty acid monolayer and a few exclusive structural proteins (Perilipin 1, 2, and 3) and are present in wide range of organisms. In mosquitoes, they are associated with lipid-storing cells like fat bodies that are crucial in mediating antiviral response. A balanced lipid environment is essential for the DENV to replicate inside mosquito cells. Thus, imbalance of lipids is a possible antiviral strategy. In a study, it was found that DENV infection induced transcription of LD biogenesis and lipid storage genes, which resulted in an increased LD level in the Aag2 cell line [67]. The classical innate immune pathways (Toll and IMD) that are activated during DENV infection were found to have a direct or indirect involvement in LD accumulation in the Aag2 cell line [68]. This indicates that LD might have an important role in mosquito immunity. On the other hand, in *Wolbachia* infected mosquito cells, cellular lipid components are used due to the lack of fatty acid synthesis genes [68]. Two *Wolbachia* strains, *wMel* and *wMelPop*, lower the level of lipidome components that are essential for DENV replication in an *Ae. albopictus* cell line (Aa23-T) [69]. Hence, both *Wolbachia* and the DENV compete for the cellular resources in co-infected cells, which significantly reduces DENV replication [63]. As LD plays dual roles (as an immune component and also as a cellular resource for the DENV), it may have a contradictory role in DENV infection in mosquitoes. 

Immune gene activation: *Wolbachia* induces a reduction–oxidation (Redox) reaction in the mosquito to produce reactive oxygen species (ROS). The oxidative stress activates the Toll pathway through which the antimicrobial peptides cecropin and defensin are produced [70]. *Wolbachia* also increases the expression of vago1 in *Ae. aegypti*, which acts as a ligand in the JAK-STAT pathway [71].

Role of *Wolbachia* in RNA interference: *Wolbachia* infection induces differential expression of host miRNAs, for example, miRNA aae-miR-2940. The aae-miR-2940 activates the metalloprotease gene, which is crucial for *Wolbachia* colonization [72]. *Wolbachia* infection downregulates AaDnmt2. AaDnmt2 is found to be overexpressed during DENV infection. So, downregulation of the AaDnmt2 during *Wolbachia* infection in *Ae. aegypti* might be a strategy by which *Wolbachia* counteracts the DENV [73].

## 8. Adaptive Immunity

Unlike vertebrates, the adaptive immunity of insects is poorly understood. In *Anopheles gambiae*, The Down syndrome cell adhesion molecule (Dscam) is found to have characteristics similar to those of antibodies found in vertebrates [74]. Insects’ adaptive immunity against RNA viruses is provided by the RNAi mechanism. During viral infection, the viral dsRNA replicative intermediate is processed by dicer2 to generate siRNA which may pass to the neighboring cell providing adaptive immunity against the virus. The viral dsRNA replicative intermediate released from apoptotic cells is taken up by the neighboring cell, which also provides adaptive immunity [75]. Recently, many endogenous non-retroviral elements (ENE) were identified in the mosquito genome. These viral sequences are acquired from previous viral infection. Transposable elements (TE) are one of the master players of genomic variation and new sequence acquisition. Among different TEs, long terminal repeat (LTR) are found predominantly upstream and downstream of the ENE. These ENEs integrate into higher density LTR loci, such as piRNA clusters, and the ENE containing piRNA clusters produce more piRNAs [76]. The piRNA generated from the ENE piRNA clusters may interact with the specific viral RNA sequences to inhibit viral replication and accumulation in the host cell. Since the piRNA clusters are stably integrated into the mosquito germ line, this type of adaptive immunity is also passed to the next generation.

## 9. What is Next?

The DENV resides as a commensal in its vector, and the physiological effects due to viral propagation in mosquitoes are different from those in the human system. 

Many signaling pathways that are active during the developmental stages of an insects are also involved in protecting insects against microbial attack. Activation of these signaling pathways results in expression of antimicrobial peptides (AMPs) that are up-regulated in virus-infected cells [24]. Generally, these AMPs target the bacterial outer membrane and cell wall biosynthesis. Viruses have significantly different structural components than bacteria, and thus the molecular mechanism of action of AMPs against the DENV needs further exploration. Similarly, small RNAs have been extensively studied, but it is still an open question as to exactly how these pathways are related to the evolutionarily conserved signaling pathways that are known to play a significant role against the DENV.

Recently, a group of researchers developed DENV-resistant mosquitoes by artificial activation of the JAK/STAT pathway [77]. These DENV resistant *Ae. aegypti* mosquitoes showed decreased capacity for egg production due to immune activation. Similar strategies of genetic engineering involving other immunity-related genes can be potentially useful methods to control the DENV [78].

Taken together, we are yet to understand the intricate details and complexity of the molecular basis of immune function of mosquitoes against the viruses that they carry. A better knowledge in this area might help open new possibilities with respect to the therapeutic intervention against mosquito-borne viral infections and vector control.
